# Influence of multiple environmental factors on the quality and flavor of watermelon juice

**DOI:** 10.1039/c9ra01533g

**Published:** 2019-05-16

**Authors:** Ye Liu, Huanlu Song, Xiao Yang, Congcong He

**Affiliations:** Beijing Engineering and Technology Research Center of Food Additives, School of Food and Chemical Engineering, Beijing Technology and Business University (BTBU) No. 11, Fucheng Road, Haidian District Beijing 100048 China liuyecau@126.com +86-010-68984547

## Abstract

Environmental factors (heat, pH, oxygen, light) can induce significant quality changes in watermelon juice during processing. To ascertain the effect of such factors on the quality of watermelon juice, the total soluble solids (TSSs), turbidity, lycopene content, color, and flavor were evaluated during different treatments. The pH had a slight impact on the content of lycopene, but had an obvious impact on turbidity. Heat, oxygen, and illumination had considerable effects on the color of watermelon juice, and the results were visible. The content of aldehydes [hexanal, nonanal, (*E*)-2-noneal, (*Z*)-6-nonenal] and ketones (6-methyl-5-hepten-2-one, geranylacetone, β-ionone) decreased in treated watermelon juices, while those of 1-nonanol, (*Z*)-3-nonen-1-ol and (*E*,*Z*)-3,6-nonadien-ol increased during illumination. The order of influence of environmental factors on watermelon-juice quality was light > pH > oxygen > heat.

## Abstract 2

Environmental factors (heat, pH, oxygen, light) can induce significant quality changes in watermelon juice during processing.

## Graphical abstract

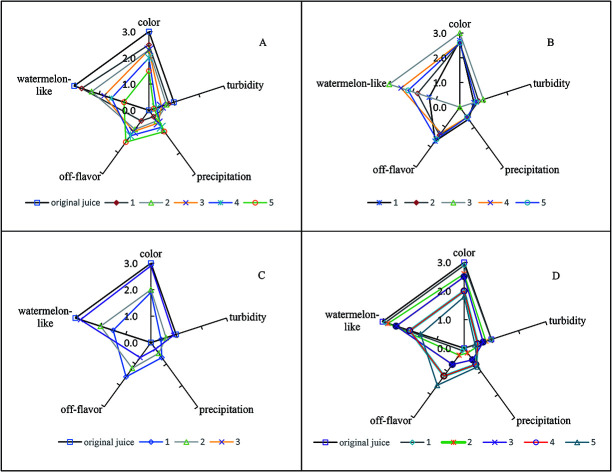

## 1. Introduction

Watermelon juice is becoming increasingly popular due to its refreshing taste, attractive color/flavor and potential health benefits.^1,2^ Watermelon contains health-promoting phytochemicals such as lycopene, β-carotene, flavonoids, phenolic compounds and vitamins.^3,4^ Phytochemicals are known to have antioxidant effects, which can protect against diabetes mellitus, cancer, hypercholesterolemia, and various other oxidative stress-induced chronic diseases.^5,6^

However, watermelon is a heat-sensitive fruit, the quality and flavor of which is affected by environmental factors. In particular, temperature and long-term exposure to the atmosphere can lead to color deterioration and loss of nutrients. Few studies have focused on the quality of watermelon juice. Watermelon juice heated at 90 °C for 60 s has higher viscosity and a higher lightness (L*) value than watermelon juice untreated for 56 days of storage.^7^ In one study, the total color difference (Δ*E*) after thermal treatments is >3.0, indicating that significant color change occurred in treated watermelon juice, and the lycopene concentration of thermal-treated watermelon juices decreased.^8^ Compared with untreated watermelon juice, the viscosity and Δ*E* increased and cloudiness decreased significantly in watermelon juices that had undergone thermal treatment, whereas the pH, total soluble solids (TSSs), titratable acidity, lycopene content, and total phenolic content did not change.^9^ In one study, lycopene levels of fresh-cut watermelon slices without rinds declined from 55.4 to 47.9 mg kg^−1^ fresh weight (FW) and the L* value increased from 43.2 to 45.8 after 2 days of storage at 4 °C, and removing rinds accelerated senescence.^10^ In addition, pasteurization at 87.7 °C for 20 s and storage for up to 30 days at 4 or 8 °C has been shown to significantly reduce the red color and levels of bioactive compounds (lycopene, antioxidant capacity and total polyphenols) of watermelon juice, particularly if the storage time is extended and a temperature of 8 °C was used.^11^ Also, ultrahigh-temperature treatment (120 and 135 °C) has been shown to inactivate microbial colonies and maintain the original color of watermelon juice, and to maintain the phenolic content by reducing polyphenol oxidase activity.^12^ Ultrahigh-temperature (135 °C for 2 s) and low-temperature long-term treatment (60 °C for 30 min) can reduce the total flora count and maintain the color of pasteurized watermelon juice, whereas the high-temperature, short-term treatment can lead to a significant color difference.^13^ The color variations observed in watermelon juice were attributed to a decrease in lycopene content (25%), as well as reductions in residual peroxidase activity (16.8%) 10 days after hyperbaric storage at 100 MPa.^14^

Some studies have focused on the flavor of watermelon or in watermelon juice. Watermelon flavor is the result of a very complex mixture of ∼71 compounds, such as aldehydes and alcohols (which dominate quantitatively) as well as ketones and furans.^15^ The most abundant compounds are thought to be hexanal, (*E*)-2-nonenal, nonanal, (*Z*)-6-nonenal, 1-nonanol, (*E*,*Z*)-2,6-nonadienal and (*Z*)-3-nonen-1-ol.^16–18^ Moreover, 6-methyl-5-hepten-2-one, geranylacetone and β-ionone have been reported to be important contributors to the unique flavor of watermelon.^15,16^ However, few reports have investigated watermelon off-flavor during processing and storage. No substantial changes have been observed in the initial content of hexanal, (*E*)-2-nonenal, nonanal, or (*Z*)-6-nonenal after thermal treatment at 90 °C for 30 s, whereas the content of 6-methyl-5-hepten-2-one, geranylacetone, 1-nonanol, and (*Z*)-3-nonen-1-ol increase slightly.^19^ Fresh-cut watermelon removing the rind has slightly perceptible off-flavor after 9 days of storage at 4 °C.^10^ Based on the mean hedonic ratings of color, flavor and overall acceptability, watermelon-juice samples stored at 4 °C for 20 days are at the limit of marketability through sensory evaluation.^11^ Conventionally, high temperature is used to inactivate microorganisms and enzymes, which leads to instability in the compounds associated with watermelon-juice flavor during juice processing and storage.^20,21^ There are 26 and 29 volatile compounds in unfermented and fermented watermelon juice, respectively. The content of 1-nonanol, 3,6-nonadien-1-ol, nonanal, (*E*)-2-nonenal, and (*E*,*Z*)-2,6-nonadienal has been reported to be reduced from 92.08 mg L^−1^ in unfermented watermelon juice to 26.41 mg L^−1^ in fermented watermelon juice.^22^ Watermelon juice treated at low temperature for a long time (60 °C for 30 min) contains the compounds associated with the aroma of watermelons, such as (*Z*)-3-nonen-1-ol, (*E*)-2-nonen-1-ol, 1-nonanal, (*E*)-2-nonenal, and (*E*,*Z*)-2,6-nonadienal, which is similar to that of unpasteurized watermelon juice.^13^

Studies have focused on the effect of processing and storage on the quality and flavor of watermelon or watermelon juice. However, the impact of heat, illumination, oxygen and pH upon watermelon juice has not been investigated systematically, a knowledge gap that we tried to bridge in the present study. Specifically, the color, turbidity, TSSs, lycopene content and changes in levels of flavor-associated compounds in watermelon juice were subjected to temperature, light, oxygen and pH treatments. We hoped to provide: (i) evaluation of the effects of thermal, illumination, oxygen and pH treatments upon the quality parameters of watermelon juice; (ii) instructions for watermelon-juice processing.

## 2. Materials and methods

### 2.1 Chemicals

*N*-Alkanes (C_6_–C_30_) used for the linear retention index (LRI) calculation were obtained from Sigma-Aldrich (Saint louis, MO, USA). All reference standards for qualitative and quantitative analyses of aromatic compounds (Table 2) were purchased from Sigma-Aldrich, Aladdin Reagents (Shanghai, China) and J&K Chemicals (Beijing, China). Hexane (HPLC grade) served as the solvent for dissolving reference standards, and 2-methyl-3-heptanone (internal standard (IS)) was purchased from Sigma-Aldrich. Sodium chloride was used for volatile extraction and other reagents were purchased from Huihai Scientific Instruments (Beijing, China).

### 2.2 Processing of watermelon juice

Watermelons were purchased from a supermarket in Beijing (China). They were washed, peeled, and crushed with a blender (HR2860; Philips, Amsterdam, the Netherlands). The juice was subjected to further analyses.

### 2.3 Thermal treatment of watermelon juice

Watermelon juice was subjected to heat processes at 50, 60, 70, 80, and 90 °C for 60 s, respectively. Watermelon juice was thermally processed in a tubular stainless-steel heat-exchange coils (internal diameter, 2.2 mm; length, 11 m) immersed in a hot-water shaking bath. After heating, the juice was cooled down immediately to 5 ± 1 °C by immersion in an ice water bath.^23^

### 2.4 Illumination treatment of watermelon juice

Illumination treatment was carried out using an illumination incubator (Jiangdong Precision Instruments, Suzhou, China) with five levels (4400, 8800, 13 200, 17 600, and 22 000 lux). The treatment temperature was maintained <25 °C to avoid the impact of a thermal effect on the quality of watermelon juice. At each level, 150 mL of watermelon juice in a 250 mL beaker was treated for 2 h. Then, the watermelon juice was stored at 4 °C in a domestic refrigerator for subsequent experiments.

### 2.5 Acid treatment of watermelon juice

The pH of 150 mL of watermelon juice was adjusted by disodium hydrogen phosphate–citric acid buffer solution at intervals of 1 pH unit from 3.5 to 7.5 with a pH meter (INESA Scientific Instruments, Shanghai, China). The pH of the original watermelon juice was 5.7 ± 0.01 (∼5.5).

### 2.6 Oxygen-free treatment of watermelon juice

Aliquots of watermelon juice (150 mL) were transferred to 860 mL plastic boxes (Lock & Lock Trade, Shanghai, China). Then, some boxes were untreated, some had half of the oxygen removed, and some had all of the oxygen removed. Oxygen removal was achieved using nitrogen under the control of a gas flowmeter. Then, the watermelon juice was stored at 4 ± 1 °C for 2 days.

### 2.7 TSS determination

Watermelon juice (0.1 mL) was allowed to drip onto a hand-held refractometer with auto-temperature compensation (PLA-1; Atago, Tokyo, Japan) after distilled water had been taken for calibration. TSS values (°Brix) were measured at 25 °C.

### 2.8 Determination of turbidity

Watermelon juice (15 mL) was transferred onto a cuvette after distilled water was removed for calibrating the equipment. The turbidity was measured using a turbidity meter (Qiwei Instruments, Hangzhou, China) at 25 °C.

### 2.9 Determination of lycopene content

Watermelon juice (2 g) was weighed. Lycopene was extracted thrice with 30, 25 and 25 mL of hexane containing 2% dichloromethane, respectively. The solvent-phase extracts were combined and diluted with the mixed solvent to 100 mL. Then, the extract underwent measurement with an UV-VIS spectrophotometer (Shimadzu, Tokyo, Japan). The lycopene content of the extract was calculated by eqn (1):^24^1
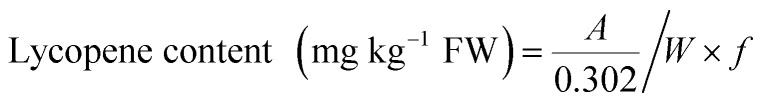
where *A* is the absorbance at 503 nm, 0.302 is the slope of the standard curve, *W* is the weight of watermelon juice, and *f* is the dilution ratio.

### 2.10 Determination of color

Color assessment of the sample was conducted randomly in reflectance mode six times using a chromameter (SK-80C; Kangguang Instruments, Beijing, China). The L*, a* and b* values of the sample were measured, and the total color difference (Δ*E*) was calculated by eqn (2):^25,26^

2 Δ*E* = [(*L* − *L*_0_)^2^ + (*a* − *a*_0_)^2^ + (*b* − *b*_0_)^2^]^1/2^

where Δ*E* is the total color difference between a sample and the control, *L* is the lightness of a sample, *L*_0_ is the lightness of the control, *a* is a redness of the sample cuvette, *a*_0_ is a redness of the control, *b* is the yellowness of a sample, and *b*_0_ is the yellowness of the control.

### 2.11 Analysis and identification of flavor-associated compounds

#### Extraction of volatiles from watermelon juice using manual solid-phase microextraction (SPME)

The major flavor-associated compounds found in the headspace of untreated and treated samples of watermelon juice were analyzed by combining solid-phase microextraction (SPME) and gas chromatography-olfactometry-mass spectrometry (GC-O-MS).^27^ Watermelon juice (10 mL) was transferred to a 40 mL vial containing 3 g of NaCl. A SPME fiber (Supelco, Bellefonte, PA, USA), coated with 50/30 μm of divinylbenzene/carboxen/polydimethylsiloxane (DVB/CAR/PDMS), was inserted into the headspace of each vial. Afterwards, the vial was heated at 50 °C for 40 min to facilitate the release of volatile compounds from the sample to the headspace volume.

#### Gas chromatography-mass spectrometry (GC-MS)

The qualitative and quantitative analyses of volatile compounds were conducted using a gas chromatograph (7890A; Agilent Technologies, Wilmington, DE, USA) coupled with a mass spectrometer (7000B series; Agilent Technologies) and desorbed for 7 min in a split/splitless GC injection port, which was equipped with an inlet linear specifically for SPME use (Agilent Technologies). Volatiles were separated on a type of fused silica capillary column (DB-Wax; 30 m × 0.25 mm i.d. × 0.25 μm; J&W Scientific, Folsom, CA, USA).

The oven temperature was initially held at 40 °C for 3 min, ramped at 5 °C min^−1^ to 200 °C, held for 3 min, ramped to 230 °C at 10 °C min^−1^, held for a further 3 min, and then increased to 250 °C for 3 min. The injection port and ionizing source were maintained at 250 and 230 °C, respectively; the carrier gas was helium and used at a flow rate of 1.2 mL min^−1^. The injector mode was splitless. The mass spectrum in electron-impact mode was generated at 70 eV. The quadrupole mass filter was used at 150 °C. Chromatograms were recorded by monitoring the total ion current in a mass range of 35–200.

### 2.12 Identification and quantification of compounds

Identification of volatile compounds was based on comparison of the GC retention index (RI) with that of authentic compounds, mass spectra (comparison with the NIST 14.0 mass spectra libraries installed in the GC-MS equipment) and odor properties. The RI was calculated as follows:3
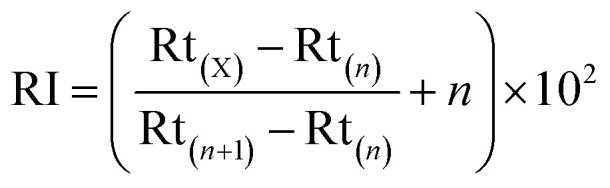
where Rt_(X)_ is the retention time of each volatile compound (X), and Rt_(*n*)_ and Rt_(*n*+1)_ are the retention times of *n*-alkanes eluting directly before and after the compound (X) under identical chromatographic conditions.

Quantitative data of the identified compounds were obtained from calculating their correction factors (CFs). The procedure of obtaining CFs was very specific. Briefly, 1 μL of 2-methyl-3-heptyl ketone (IS) was added to the mixed standard solvent, which was also added to watermelon juice. Under the same condition, CFs were calculated from the ratio of each peak area to the peak area of the IS. The concentration of the volatile compound was determined from the peak area of the IS and the volatile compound of watermelon juice based on CFs. The equations were as follows:4
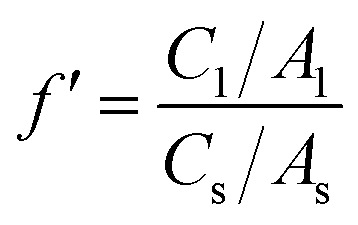
5
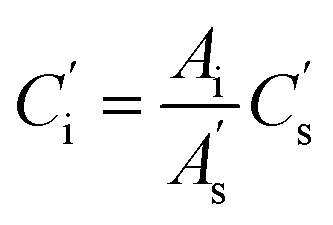
6
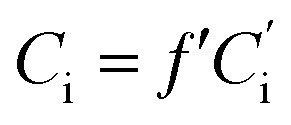
where *f*′ is a correction factor, *C*_1_ is the concentration of standard compounds in the standard solvent, *A*_1_ is the peak area of standard compounds in the standard solvent, *C*_s_ is the concentration of the IS in the standard solvent, *A*_s_ is the peak area of the IS in the standard solvent, *A*_i_ is the peak area of the component to be measured in the sample solvent, 
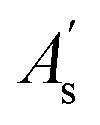
 is the peak area of the IS in the sample solvent, 
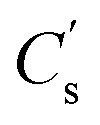
 is the concentration of the IS, 
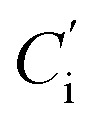
 is the concentration of the component to be measured in the sample solvent, and *C*_i_ is the concentration of the component to be measured after correction.

### 2.13 Sensory evaluation

Fifteen panelists (seven men and eight women, 22–36 years) trained in descriptive analyses were recruited from the Laboratory of Molecular Sensory Science within Beijing Technology and Business University (Beijing, China). All samples were prepared, served and evaluated under appropriate conditions. To train the sensory panel to recognize the aromatic character of watermelon, daily training sessions were conducted for 3 months. All samples of treated watermelon juice and control watermelon juice were served in randomly numbered plastic cups on a tray with a cup of water and a piece of non-salted cracker. The panelists were asked to rate their preference of “watermelon-like”, “off-flavor”, “color”, “precipitation”, and “turbidity”, from a scale from 0 to 3, respectively, where higher numbers represent a higher preference of attributes, expect for precipitation and off-flavor.^28^

### 2.14 Data analyses

Analysis of variance (ANOVA) and Duncan's multiple range tests were carried out using SAS (SAS Institute, Cary, NC, USA). The ANOVA test was carried out for all experimental runs to determine significance at 95% confidence intervals. All experiments were undertaken in triplicate.

## 3. Results and discussion

### 3.1 Effect of thermal treatment on the quality of watermelon juice

The effect of thermal treatments on TSSs, turbidity, lycopene content and color of watermelon juice are shown in Table 1. No significant differences were observed in TSSs after thermal treatments (*p* > 0.05). In contrast, the turbidity increased up to 39% after thermal treatment at 80 °C for 60 s and 33% at 90 °C for 60 s (*p* < 0.05). This might have been caused by the growth of suspended particles in the heated watermelon juice. The turbidity of fruit juices increase with increases in concentrations of polysaccharides and proteins.^29^ Active protein–polyphenol complexes grow into large colloid particles in fruit juices.^30^

**Table 1:** Effect of thermal processing on the quality parameters and flavor-associated compounds in watermelon juice

	OJ^a^	50 °C	60 °C	70 °C	80 °C	90 °C
**Quality parameters^c^**						
Soluble solid (°Brix)	8.00 ± 0.05^a^	8.00 ± 0.1^a^	7.90 ± 0.06^a^	8.00 ± 0.06^a^	8.00 ± 0.00^a^	8.00 ± 0.06^a^
Turbidity (NTU)	69.30 ± 0.52^a^	73.50 ± 0.10^b^	81.40 ± 1.57^c^	82.20 ± 0.86^c^	96.30 ± 1.76^e^	92.50 ± 0.97^d^
Lycopene (μg g^−1^)	23.31 ± 0.10^b^	26.84 ± 0.07^c^	28.3 ± 0.06^d^	35.91 ± 0.13^f^	35.37 ± 0.10^e^	21.5 ± 0.14^a^
L*	20.53 ± 0.12^c^	20.89 ± 0.20^d^	20.56 ± 0.19^c^	18.63 ± 0.04^b^	18.52 ± 0.21^b^	17.91 ± 0.18^a^
a*	20.08 ± 0.22^f^	19.02 ± 0.09^e^	18.55 ± 0.30^d^	18.14 ± 0.33^c^	17.5 ± 0.10^b^	17.12 ± 0.14^a^
b*	20.16 ± 0.24^c^	20.54 ± 0.32^d^	19.99 ± 0.32^c^	19.35 ± 0.07^b^	18.86 ± 0.07^a^	18.76 ± 0.16^a^
Δ*E*	—	1.18	1.54	2.83	3.52	4.19

**Compounds (ng mL** ^**−1**^ **)^c^**						
Hexanal	34.63 ± 0.45^c^	25.82 ± 1.57^a^	31.48 ± 1.35^b^	108.59 ± 1.45^f^	105.05 ± 1.12^e^	83.49 ± 0.79^d^
6-Methyl-5-hepten-2-one	32.69 ± 1.02^c^	16.46 ± 0.64^a^	22.34 ± 1.19^b^	55.42 ± 0.79^e^	55.08 ± 1.40^e^	46.75 ± 0.90^d^
Nonanal	52.08 ± 0.35^b^	35.43 ± 0.86^a^	55.95 ± 0.60^c^	102.79 ± 1.80^f^	78.99 ± 1.26^e^	59.23 ± 0.73^d^
(*Z*)-6-Nonenal	40.14 ± 0.72^b^	26.80 ± 0.88^a^	41.52 ± 0.93^b^	55.96 ± 1.33^d^	56.23 ± 1.34^d^	50.92 ± 3.58^c^
(*E*)-2-Nonenal	211.71 ± 3.05^e^	74.46 ± 1.59^b^	119.78 ± 1.73^c^	163.97 ± 2.49^d^	119.70 ± 0.59^c^	62.75 ± 0.78^a^
(*E*,*Z*)-2,6-Nonadienal	197.86 ± 3.47^f^	55.65 ± 0.62^a^	92.18 ± 0.77^c^	123.09 ± 1.95^e^	100.45 ± 1.59^d^	62.18 ± 1.28^b^
1-Nonanol	15.48 ± 1.59^a^	48.39 ± 0.62^b^	72.18 ± 1.23^d^	92.64 ± 2.14^f^	78.18 ± 1.05^e^	65.23 ± 1.36^c^
(*Z*)-3-Nonen-1-ol	186.02 ± 10.75^a^	401.09 ± 4.34^b^	551.29 ± 3.09^c^	707.44 ± 3.78	655.20 ± 3.98^e^	566.53 ± 5.26^f^
(*E*,*Z*)-3,6-Nonadien-1-ol	104.28 ± 2.77^a^	228.75 ± 2.92^b^	332.49 ± 2.46^c^	403.04 ± 2.35^e^	403.59 ± 2.66^e^	363.31 ± 5.67^d^
Geranylacetone	48.39 ± 1.26^e^	18.13 ± 0.44^a^	32.07 ± 1.40^b^	57.18 ± 1.46^f^	46.27 ± 0.83^d^	39.29 ± 1.02^c^
β-Ionone	6.65 ± 0.40^d^	2.66 ± 0.45^b^	4.68 ± 0.45^c^	0.89 ± 0.08^a^	ND^b^	ND

a OJ: original juice, the juice was squeezed when the parameters were determined.

b ND: not detected.

c The different letters in the same column represent the significant difference (*p* < 0.05).

The content of lycopene increased before decreasing during treatment at 50–90 °C, and the maximum lycopene content appeared at 80 °C. One could speculate that the temperature rise increased the extraction yield of the lycopene.^31^ A high temperature might damage the structure of lycopene, which would lead to the lycopene content decreasing at 90 °C.^8^ Usually, it is assumed that the color changes significantly if Δ*E* > 3, and a higher Δ*E* indicates a greater color change. Hence, high-temperature (>70 °C) treatment of watermelon juice led to a significant color change. The color of watermelon juice changed with increasing temperature. The effect of thermal treatments on the color of strawberry and pepper are similar to those on watermelon juice in the present study.^32,33^ The a* value of watermelon juice decreased significantly with increasing temperature (*p* < 0.05), which was related to redness (the main color of watermelon juice).^8^ Lycopene is the major carotenoid imparting the red color in watermelon. It has been reported that the a* value and lycopene content are positively correlated in tomatoes.^34^ However, a different result was shown in our study, which might have been due to lycopene accumulation after thermal treatment. As a whole, the b* value of watermelon juice also decreased with increasing temperature (*p* < 0.05), which was related to yellowness (which is contributed mainly by β-carotene).^35^ β-Carotene is a type of carotenoid that is degraded readily by heating.^36^ Hence, the reduction in the b* value might have been induced by a loss of β-carotene. Thermal treatments resulted in a decrease in the L* value of watermelon juice, indicating that the color became darker, which could be correlated with non-enzymatic browning. During thermal treatment, fructose and glucose can be dehydrated by acids to form hydroxymethylfurfural (HMF), which results in the browning of fruit juices or fruit purees.^37,38^ HMF is the most important chemical substance produced in non-enzymatic browning processes. It is one of the most widely used indices for studies of non-enzymatic browning in fruit juices and fruit derivatives.

The effect of temperature on the aromatic compounds in watermelon juice is shown in Table 1. The C_9_ aldehydes and alcohols identified in watermelon juice are formed enzymatically from unsaturated C_18_ fatty acids.^15^ The concentration of (*E*)-2-nonenal and (*E*,*Z*)-2,6-nonadienal in the control watermelon juice was significantly decreased, and just 30% of the concentration of (*E*)-2-nonenal was retained from the original watermelon juice after thermal treatment (90 °C for 60 s). The concentration of 1-nonanol, (*Z*)-3-nonen-1-ol and (*E*,*Z*)-3,6-nonadien-1-ol in thermally treated watermelon juice was higher than that of untreated watermelon juice. The concentration of 1-nonanol increased to 3–6-times, and (*Z*)-3-nonen-1-ol and (*E*,*Z*)-3,6-nonadien-1-ol both to 2–4 times. The concentration increase in these three compounds induced a flavor change in watermelon juice. These three compounds might have been converted by their corresponding aldehydes under the action of alcohol dehydrogenase. The flavor of watermelon juice became weak due to the higher levels of alcohols than those of aldehydes. Moreover, the content of geranylacetone and β-ionone changed (especially β-ionone). At 80 °C and 90 °C, β-ionone could not be detected by MS.

### 3.2 Effect of pH on the quality of watermelon juice

The effect of pH on TSSs, turbidity, lycopene content and color of watermelon juice is shown in Table 2. TSS levels decreased significantly when pH changed, and the minimum value appeared at pH 3.5. This result was in accordance with a study showing that lower pH values of flash-heated lime juice (FHLJ) and hot clarified juice (CJ) caused higher sucrose losses.^39^ The turbidity showed different changes in all pH treatments. At pH 3.5, the turbidity showed the biggest change, increasing up to 116.1 NTU. In neutral or alkaline conditions, the turbidity dropped 23%, which was the opposite effect seen in acidic conditions. This result may have been because quinone is formed faster as a protonated form than as a neutral from.^40^ Similarly, the particle size of milk in lime-treated FHLJ tends to be larger than that for saccharate-treated FHLJ, which causes the turbidity of juices to decrease at alkaline conditions.^39^

**Table 2:** Effect of pH on the quality parameters and flavor-associated compounds of watermelon juice

	pH 3.5	pH 4.5	pH 5.7 (OJ^a^)	pH 6.5	pH 7.5
**Quality parameters^b^**					
Soluble solid (°Brix)	6.70 ± 0.13^a^	7.40 ± 0.08^bc^	8.00 ± 0.05^d^	7.60 ± 0.00^c^	7.50 ± 0.00^b^
Turbidity (NTU)	116.10 ± 2.59^d^	112.40 ± 2.56^d^	69.30 ± 0.52^a^	97.50 ± 1.28^c^	71.10 ± 0.51^b^
Lycopene (μg g^−1^)	21.43 ± 0.24^a^	21.29 ± 0.32^a^	23.31 ± 0.10^b^	21.96 ± 0.49^a^	21.80 ± 0.37^a^
L*	15.58 ± 0.33^c^	11.83 ± 0.47^a^	20.53 ± 0.12^e^	13 ± 0.25^b^	17.16 ± 0.30^d^
a*	17.77 ± 0.30^a^	19.6 ± 0.67^b^	20.08 ± 0.22^c^	22.81 ± 0.28^d^	23.61 ± 0.09^e^
b*	18.71 ± 0.13^a^	19.49 ± 0.57^a^	20.16 ± 0.24^b^	21.8 ± 1.15^c^	23.89 ± 0.10^d^
Δ*E*	5.36	5.10	—	2.70	3.13

**Compounds (ng mL** ^**−1**^ **)^b^**					
Hexanal	4.74 ± 0.09^a^	5.16 ± 0.23^a^	34.63 ± 0.45^c^	7.18 ± 0.34^b^	ND
6-Methyl-5-hepten-2-one	22.55 ± 1.15^b^	20.71 ± 1.29^ab^	32.69 ± 1.02^c^	20.27 ± 0.91^ab^	19.01 ± 1.14^a^
Nonanal	24.16 ± 1.42^b^	7.69 ± 0.13^a^	52.08 ± 0.35^c^	22.72 ± 0.84^b^	8.35 ± 0.70^a^
(*E*)-2-Nonenal	45.29 ± 1.47^c^	22.03 ± 1.24^a^	211.71 ± 3.05^e^	70.25 ± 1.09^d^	36.32 ± 1.01^b^
(*E*,*Z*)-2,6-Nonadienal	18.23 ± 0.88^c^	12.01 ± 1.14^a^	197.86 ± 3.47^e^	27.54 ± 0.56^d^	15.33 ± 0.75^b^
1-Nonanol	54.92 ± 1.27^b^	86.82 ± 1.49^c^	15.48 ± 1.59^a^	132.77 ± 1.61^e^	112.89 ± 2.12^d^
(*Z*)-3-Nonen-1-ol	667.62 ± 6.59^b^	986.05 ± 4.46^c^	186.02 ± 10.75^a^	1127.05 ± 1.50^d^	1127.54 ± 7.90^d^
(*E*,*Z*)-3,6-Nonadien-1-ol	359.18 ± 2.47^b^	558.62 ± 1.48^c^	104.28 ± 2.77^a^	640.76 ± 1.48^d^	648.40 ± 4.26^e^
Geranylacetone	30.30 ± 0.72^c^	18.50 ± 0.90^a^	48.39 ± 1.26^d^	30.12 ± 1.22^c^	26.37 ± 0.50^b^
β-Ionone	4.45 ± 0.36^b^	3.23 ± 0.28^a^	6.65 ± 0.40^d^	5.02 ± 0.38^c^	2.79 ± 0.27^a^

a OJ: original juice, the juice was squeezed when the parameters were determined.

b The different letters in the same column represent the significant difference (*p* < 0.05).

No changes in lycopene content were observed at different pH values. Lycopene is stable under pH changes, and lycopene isomerization is a reversible reaction. Under the pH in the stomach, the all-*trans* isomer is more stable than the 13-*cis* isomer, but the total lycopene content does not change.^41^ Under a pH 3.5 and 4.5, the a* value decreased slightly, which was in accordance with the variation in lycopene content. Similarly, the b* value was reduced, and they all contributed to the Δ*E*. Under these two pH values, Δ*E* was >3.0, indicating that the color had changed significantly. The ranking of Δ*E* was pH 3.5 > pH 4.5 > pH 7.5 > pH 6.5.

The effect of pH on the aromatic compounds of watermelon juice are shown in Table 2. The concentration of hexanal decreased dramatically after acid treatments. In an alkaline condition (pH 7.5), hexanal was not detected. The content of nonanal, (*E*)-2-nonenal and (*E*,*Z*)-2,6-nonadienal decreased. Also, 13% of (*E*)-2-nonenal and 8% of (*E*,*Z*)-2,6-nonadienal in the original watermelon juice was retained at pH 3.5. On the contrary, the content of 1-nonanol, (*Z*)-3-nonen-1-ol and (*E*,*Z*)-3,6-nonadien-ol showed a large increase. Among them, the (*Z*)-3-nonen-1-ol level was 2.6-times that of untreated watermelon juice. Moreover, the geranylacetone level showed a little increase except for pH 4.5. The 6-methyl-5-hepten-2-one level also decreased. The flavor of watermelon juice also became weak, which was in accordance with the results of sensory evaluations.

### 3.3 Effect of oxygen on the quality of watermelon juice

The effect of oxygen on TSSs, turbidity, lycopene content and the color of watermelon juice is shown in Table 3. TSSs and turbidity increased slightly after oxygen-elimination treatments. The turbidity of untreated watermelon juice was 1.3-times that of fresh watermelon juice. This might have been mainly due to the condensation of tannins in watermelon juice. Condensed tannins cause turbidity by oxidation polymerization/aggregation with proteins.^42^

**Table 3:** Effect of oxygen on the quality parameters and flavor-associated compounds of watermelon juice

	OJ^a^	UJ^a^	EHO^b^	EAO^b^
**Quality parameters^c^**				
Soluble solid (°Brix)	8.00 ± 0.05^a^	8.47 ± 0.06^b^	8.53 ± 0.06^b^	8.53 ± 0.06^b^
Turbidity (NTU)	69.30 ± 0.52^a^	88.10 ± 4.47^c^	79.83 ± 5.02^b^	72.07 ± 3.10^a^
Lycopene (μg g^−1^)	23.31 ± 0.10^a^	11.09 ± 0.44^d^	17.35 ± 0.28^b^	18.86 ± 0.11^c^
L*	20.53 ± 0.12^c^	14.73 ± 0.32^a^	17.66 ± 0.39^b^	20.62 ± 0.97^c^
a*	20.08 ± 0.22^c^	18.04 ± 0.16^a^	18.95 ± 0.39^b^	19.08 ± 0.25^b^
b*	20.16 ± 0.24^c^	18.29 ± 0.48^a^	18.82 ± 0.33^ab^	19.41 ± 0.32^b^
Δ*E*	—	6.42	3.36	1.25

**Compounds (ng mL** ^**−1**^ **)^c^**				
Hexanal	34.63 ± 0.45^c^	7.43 ± 3.66^a^	12.55 ± 2.15^b^	15.07 ± 0.19^b^
6-Methyl-5-hepten-2-one	32.69 ± 1.02^a^	25.59 ± 4.16^a^	31.49 ± 4.66^a^	21.60 ± 3.87^a^
Nonanal	52.08 ± 0.35	37.27 ± 3.77	38.84 ± 0.49	54.52 ± 1.58
(*Z*)-6-Nonenal	40.14 ± 0.72^c^	6.59 ± 0.20^a^	9.13 ± 2.41^a^	26.43 ± 3.94^b^
(*E*)-2-Nonenal	211.71 ± 3.05^c^	148.03 ± 49.07^a^	193.26 ± 1.46^b^	238.88 ± 15.81^c^
(*E*,*Z*)-2,6-Nonadienal	197.86 ± 3.47^c^	41.73 ± 9.81^a^	39.44 ± 2.91^a^	114.89 ± 0.89^b^
1-Nonanol	15.48 ± 1.59^a^	123.93 ± 0.67^b^	145.68 ± 8.37^c^	131.42 ± 27.67^bc^
(*Z*)-3-Nonen-1-ol	186.02 ± 10.75^a^	521.64 ± 44.24^bc^	636 ± 20.08^c^	570.59 ± 38.05^b^
(*E*,*Z*)-3,6-Nonadien-1-ol	104.28 ± 2.77^a^	247.26 ± 7.23^b^	348.36 ± 9.96^c^	267.13 ± 12.24^bc^
Geranylacetone	48.39 ± 1.26^a^	75.12 ± 2.69^b^	83.51 ± 3.67b^c^	93.09 ± 12.23^c^
β-Ionone	6.65 ± 0.40^ab^	6.33 ± 0.92^a^	7.27 ± 1.45^ab^	7.89 ± 0.66^b^

a OJ: original juice, the juice was squeezed when the parameters were determined. UJ: untreated juice: the juice did not eliminate the oxygen, and was stored with EHO and EAO.

b EHO: eliminated half the oxygen. EAO: eliminated all the oxygen.

c The different letters in the same column represent the significant difference (*p* < 0.05).

The concentration of lycopene also decreased. In untreated watermelon juice, the lycopene content decreased 52%. The lycopene content was 19% in watermelon juice in which all oxygen had been eliminated, which might have been caused by the oxidation of lycopene. Elimination of 50% of oxygen and 100% of oxygen led to significant color changes because Δ*E* after each treatment was >3.0. The ranking of Δ*E* was untreated watermelon juice >50%-eliminated-oxygen watermelon juice >100%-eliminated-oxygen watermelon juice. Hence, oxygen had an important influence on the color of watermelon juice. The L* value also decreased, and the color of the watermelon was no longer attractive. This might have been due to oxidative browning *via* vitamin C, and oxygen is essential for enzymatic browning.^43^

The effect of oxygen on the aromatic compounds of watermelon juice is shown in Table 3. The content of hexanal and 6-methyl-5-hepten-2-one declined. Moreover, the hexanal content in untreated watermelon juice retained only 21% that of fresh watermelon juice. The (*Z*)-6-nonenal content also decreased and, in untreated watermelon juice, it retained only 16% that of fresh watermelon juice. These data suggested that oxygen had important effects on these compounds. In addition, the concentration of nonanal and (*E*)-2-nonenal decreased in untreated watermelon juice and 50%-eliminated-oxygen watermelon juice, but did not change in 100%-eliminated-oxygen watermelon juice. Levels of geranylacetone and β-ionone decreased in untreated watermelon juice. The content of 1-nonanol, (*Z*)-3-nonen-1-ol and (*E*,*Z*)-3,6-nonadien-ol also decreased in untreated watermelon one.

### 3.4 Effect of light on the quality of watermelon juice

The effect of light on TSSs, turbidity, lycopene content and the color of watermelon juice is shown in Table 4. TSSs content increased with increasing illumination intensity. The turbidity also increased. Fruit quality (weight, hardness, TSSs) has been shown to be positively correlated with the relative light intensity.^44^

**Table 4:** Effect of illumination on the quality parameters and flavor-associated compounds of watermelon juice

	OJ^a^	4400 lux	8800 lux	13 200 lux	17 600 lux	22 000 lux
**Quality parameters** b						
Soluble soild (°Brix)	8.00 ± 0.05^a^	10.00 ± 0.07^b^	10.20 ± 0.43^b^	10.50 ± 0.09^c^	10.80 ± 0.15^d^	10.70 ± 0.25^bc^
Turbidity (NTU)	69.30 ± 0.52^a^	89.20 ± 2.39^b^	91.30 ± 3.26^b^	93.20 ± 0.82^b^	100.00 ± 2.99^c^	110.00 ± 2.34^d^
Lycopene (μg g^−1^)	23.31 ± 0.10^a^	31.90 ± 0.11^d^	31.15 ± 0.1^c^	30.50 ± 0.06^c^	25.67 ± 0.31^b^	39.04 ± 0.8^e^
L*	20.53 ± 0.12^d^	18.20 ± 0.35^c^	17.70 ± 1.86^ab^	15.81 ± 0.18^a^	15.13 ± 1.28^abc^	14.93 ± 0.94^bc^
a*	20.08 ± 0.22^c^	18.40 ± 0.16^b^	18.38 ± 0.16^b^	17.62 ± 0.34^a^	17.74 ± 0.61^a^	17.43 ± 0.7^a^
b*	20.16 ± 0.24^a^	21.00 ± 0.30^b^	21.49 ± 0.67^b^	19.74 ± 0.46^a^	21.63 ± 0.73^b^	21.54 ± 0.4^b^
Δ*E*	—	2.97	3.54	5.31	6.04	6.32

**Compounds (ng mL** ^**−1**^ **)^b^**						
Hexanal	34.63 ± 0.45^e^	13.19 ± 0.79^cd^	16.16 ± 1.5^d^	4.28 ± 1.27^bc^	3.90 ± 1.29^b^	2.20 ± 0.67^a^
6-Methyl-5-hepten-2-one	32.69 ± 1.02^e^	13.43 ± 1.31^cd^	14.77 ± 2.34^d^	11.88 ± 1.08^bc^	10.05 ± 2.90^b^	3.81 ± 0.02^a^
Nonanal	52.08 ± 0.35^e^	13.02 ± 1.62^cd^	15.27 ± 0.64^d^	12.44 ± 0.62^bc^	8.83 ± 3.40^b^	4.05 ± 0.27^a^
(*Z*)-6-Nonenal	40.14 ± 0.72^e^	10.87 ± 1.06^d^	9.00 ± 0.26^c^	3.72 ± 0.14^b^	3.59 ± 1.10^b^	1.89 ± 0.08^a^
(*E*)-2-Nonenal	211.71 ± 3.05^c^	328.37 ± 10.07^e^	268.01 ± 5.38^d^	155.47 ± 18.00^b^	48.18 ± 15.72^a^	47.95 ± 2.66^a^
(*E*,*Z*)-2,6-Nonadienal	197.86 ± 3.47^d^	206.92 ± 7.47^e^	164.32 ± 2.35^c^	64.73 ± 11.16^b^	18.28 ± 5.27^a^	26.36 ± 1.10^a^
1-Nonanol	15.48 ± 1.59^b^	6.67 ± 0.36^a^	13.89 ± 2.86^b^	48.46 ± 1.22^c^	44.82 ± 12.55^c^	6.84 ± 0.45^a^
(*Z*)-3-nonen-1-ol	186.02 ± 10.75^b^	128.03 ± 0.44^a^	261.51 ± 17.60^c^	364.78 ± 39.15^d^	339.53 ± 60.60^d^	93.29 ± 6.74^a^
(*E*,*Z*)-3,6-Nonadien-1-ol	104.28 ± 2.77^b^	65.06 ± 4.77^a^	152.75 ± 11.19^c^	253.24 ± 3.08^d^	230.23 ± 43.18^d^	74.35 ± 3.07^a^
Geranylacetone	48.39 ± 1.26^e^	17.54 ± 1.38^c^	20.22 ± 3.77^d^	13.36 ± 0.26^b^	10.78 ± 2.88^b^	4.85 ± 0.43^a^
β-Ionone	6.65 ± 0.40d	1.81 ± 0.35^bc^	2.20 ± 0.52^c^	1.43 ± 0.24^b^	1.32 ± 0.49^b^	0.41 ± 0.03^a^

a OJ: original juice: the juice was squeezed when the parameters were determined.

b The different letters in the same column represent the significant difference (*p* < 0.05).

The lycopene content in treated watermelon juice was more than that of untreated watermelon juice. However, it decreased with increasing illumination intensity except for that at 22 000 lux. According to the Δ*E* value, the color changed significantly in an illumination intensity of 8800–22 000 lux. The fact that Δ*E* increased with increasing illumination intensity suggested that illumination had a significant influence on color. The decreasing values of L* suggested the color became darker. Moreover, the a* value of treated watermelon juice was lower than that of untreated watermelon juice, which suggested that the treated watermelon juice became more yellow and less red. This might have been caused by oxidation of vitamin C under illumination.^45^

The effect of light on the aromatic compounds in watermelon juice is shown in Table 4. The content of hexanal and 6-methyl-5-hepten-2-one both decreased obviously. The photo-oxidation of lipids in watermelon juice can lead to production of alcohols, aldehydes, acids and esters. First, the lipids resolve into aldehydes, and then the aldehydes are transformed to alcohols, and this explains why the levels of aldehydes decrease, but the levels of alcohols increase, with increasing illumination intensity.^46^ Hence, the content of nonanal and (*Z*)-6-nonenal decreased with increasing illumination intensity. The (*E*)-2-nonenal content increased slightly at 4400 and 8800 lux but, with increasing light intensity, it decreased. The content of 1-nonanol, (*Z*)-3-nonen-1-ol and (*E*,*Z*)-3,6-nonadien-ol decreased after increasing initially. At 22 000 lux, levels of all the aromatic compounds changed significantly, and the aroma of the watermelon worsened.

### 3.5 Sensory evaluation of watermelon juice

**Fig. 1:**
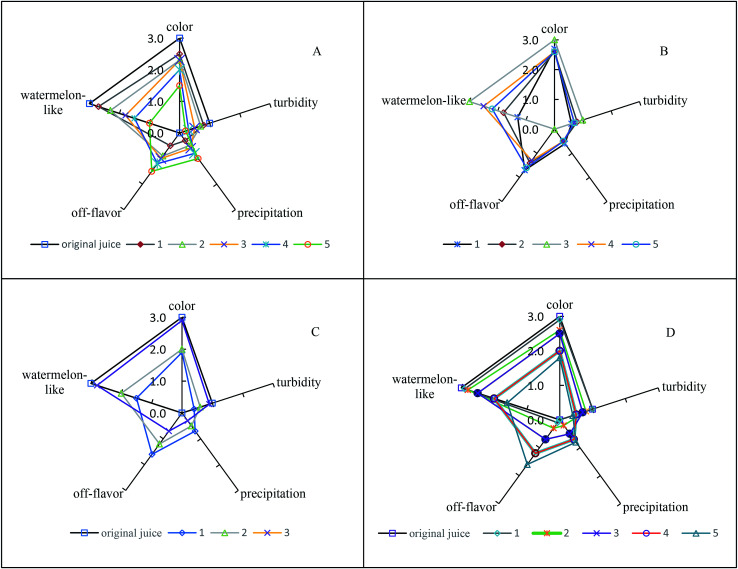
Effect of environmental factors on the sensory quality of watermelon juice: (A) no. 1: 50 °C for 60 s, no. 2: 60 °C for 60 s, No. 3: 70 °C for 60 s, no. 4: 80 °C for 60 s, no. 5: 90 °C for 60 s; (B) no. 1: pH 3.5, no. 2: pH 4.5, no. 3: pH 5.7 (untreated watermelon juice), no. 4: pH 6.5, no. 5: pH 7.5; (C) no. 1: untreated watermelon juice, no. 2: 50%-eliminated oxygen, no. 3: 100%-eliminated oxygen; (D) 1: 4400 lux, 2: 8800 lux, 3: 13 200 lux, 4: 17 600 lux, 5: 22 000 lux.

The results of sensory evaluation after thermal, pH, oxygen, and illumination treatments are shown in Fig. 1. After thermal treatment, the watermelon-like flavor decreased with increasing temperature, and the off-flavor increased. Moreover, the color changed significantly. Therefore, thermal treatments had important effects on the quality and flavor of watermelon juice. When the pH of watermelon juice was adjusted to 3.5, the off-flavor was very obvious, especially the pungent odor. The other attributes (color, turbidity, precipitation) also changed. The sensory quality of watermelon juice had a significant negative relationship with the oxygen content. Oxygen caused nonenzymatic browning reactions, which had a deleterious effect on the color of watermelon juice. Peroxidase initiated a series of reactions in the presence of atmospheric oxygen, and the flavor and appearance of watermelon juice worsened.^47^ After illumination treatment, the sensory quality worsened with increasing illumination intensity. Illumination promoted the browning of watermelon juice.

### 3.6 Regression analysis of environmental factors and quality parameters

The standard regression coefficients of environmental factors on the quality parameters of watermelon juice are shown in Table 5. Positive values denote that the quality parameters increased with increasing environmental factors, and *vice versa*. All the environmental factors tested affected the quality parameters of watermelon juice. Hence, absolute values were taken to calculate the sum of standard regression coefficients. A higher sum value denoted a great impact of the environmental factor on the quality of watermelon juice. The order of factors was illumination > pH > oxygen > heat.

**Table 5:** Standard regression coefficients of environment factors on the quality parameters of watermelon juice^a^

Factors	Soluble solid	Turbidity	Lycopene	L*	a*	b*	Sum (absolute value)
Thermal	—	—	0.468	−0.149	−0.608	−0.380	1.605
pH	0.159	−0.594	—	0.376	0.589	0.670	2.388
Oxygen	−0.209	0.194	−0.332	−0.251	0.317	0.372	1.675
Illumination	0.883	0.364	0.648	−0.423	−0.598	0.146	3.062

a “—”: this factor did not impact this quality parameter significantly.

## 4. Conclusions

Environmental factors during treatment (heat, pH, oxygen and light) had a significant impact on the color, TSSs, turbidity, lycopene content and flavor-associated compounds of watermelon juice. The extent of this impact was verified by sensory evaluation. Through regression analysis, the order of factors that influenced watermelon-juice quality was light > pH > oxygen > heat. These results provide: (i) a basis to solve the problem of quality deterioration during high-temperature sterilization; (ii) new ways to preserve and improve the valuable attributes of watermelon juice.

## Conflicts of interest

The authors declared there are no conflicts of interest.

## Supplementary Material
